# Materia Medica, Pharmacology and Therapeutics

**Published:** 1890-01

**Authors:** 


### Reviews.

"Materia Medica, Pharmacology and Therapeutics."—Shoemaker & Auldi. F. A. Davis, Publisher.

The first volume of this work has just been received.

From a personal acquaintance with one of the authors, we had been led to expect a pains taking disertation—up to the times, bearing evidence of careful and thorough preparation, introducing new features, and discarding that which has served for so long, just to add to the bulk. We have not been disappointed, for from a partial examination—fragmentary, because when any subject treated in the modest volume was begun, we could not skip, so great was our attention—interrupted by arduous professional duties. We gladly welcome it to our shelves, and predict for it a permanent and lasting popularity.

We sincerely hope that the 2d vol. will not be long delayed, as we are persuaded if it keeps up to the high standard of the 1st, that it will be a rival of such works as Biddle or Bartholow for popularity among medical studants that will cause them to look well to their laurels.

"Oh! that my enemy would write a book" cannot be said of Shoemaker & Auldi. J. S. T.

